# Identification of alcohol stress tolerance genes of *Synechocystis* sp. PCC 6803 using adaptive laboratory evolution

**DOI:** 10.1186/s13068-017-0996-5

**Published:** 2017-12-20

**Authors:** Takuya Matsusako, Yoshihiro Toya, Katsunori Yoshikawa, Hiroshi Shimizu

**Affiliations:** Department of Bioinformatic Engineering, Graduate School of Information Science and Technology, Osaka University, 1-5 Yamadaoka, Suita, Osaka 565-0871 Japan

**Keywords:** *Synechocystis* sp. PCC 6803, Alcohol stress tolerance, Adaptive laboratory evolution, Isobutanol

## Abstract

**Background:**

*Synechocystis* sp. PCC 6803 is an attractive organism for the production of alcohols, such as isobutanol and ethanol. However, because stress against the produced alcohol is a major barrier for industrial applications, it is highly desirable to engineer organisms with strong alcohol tolerance.

**Results:**

Isobutanol-tolerant strains of *Synechocystis* sp. PCC 6803 were obtained by long-term passage culture experiments using medium containing 2 g/L isobutanol. These evolved strains grew on medium containing 5 g/L isobutanol on which the parental strain could not grow. Mutation analysis of the evolved strains revealed that they acquired resistance ability due to combinatorial malfunctions of slr1044 (*mcpA*) and slr0369 (*envD*), or slr0322 (*hik43*) and *envD*. The tolerant strains demonstrated stress resistance against isobutanol as well as a wide variety of alcohols such as ethanol, *n*-butanol, and isopentanol. As a result of introducing an ethanol-producing pathway into the evolved strain, its productivity successfully increased to 142% of the control strain.

**Conclusions:**

Novel mutations were identified that improved the stress tolerance ability of various alcohols in *Synechocystis* sp. PCC 6803.

**Electronic supplementary material:**

The online version of this article (10.1186/s13068-017-0996-5) contains supplementary material, which is available to authorized users.

## Background

Cyanobacteria are promising industrial microorganisms for bio-production because the cells can directly fix atmospheric carbon dioxide and convert it to a target compound using energy from photosynthesis. In recent years, the cyanobacterial production of alcohols, such as ethanol, *n*-butanol, and isobutanol, as an alternative to petroleum has attracted attention [1]. Isobutanol is an attractive fuel because of its higher energy density and lower hygroscopicity than ethanol. Overexpression of ribulose 1,5-bisphosphate carboxylase/oxygenase genes has led to the production of 450 mg/L isobutanol production in *Synechococcus elongatus* PCC 7942 [2]. However, its productivity is still lower than that of fermentation microorganisms such as *Escherichia coli* and *Corynebacterium glutamicum* [3, 4]. Alcohol stress is one of the causes of low productivity in cyanobacteria. In isobutanol production of *Synechocystis* sp. PCC 6803, production of biomass and isobutanol increased about 1.2 and 2.5 times, respectively, by in situ removal of the produced isobutanol using a solvent trap [5]. Because the growth reduction caused by alcohol stress occurs at a low concentration of 200 mg/L isobutanol, it is highly desirable to engineer cyanobacterial strains with strong alcohol tolerance for industrial production of isobutanol.

In a previous study, transcriptome responses of *Synechocystis* sp. PCC 6803 to 1-butanol identified candidate genes for enhancing 1-butanol tolerance, and the expression of small heat-shock protein HspA (sll1514) and hypothetical protein CccS (slr1617) successfully improved 1-butanol tolerance [6]. Furthermore, screening from the response regulator library revealed that sll0039 and slr1037 are involved in 1-butanol tolerance in *Synechocystis* sp. PCC 6803 [7, 8]. The co-overexpression of slr1037 and sll0039 improved 1-butanol tolerance by 133% [9]. It has also been reported that an overexpression of sigB, which encoding RNA polymerase sigma factor, improved 1-butanol tolerance [10].

Adaptive laboratory evolution (ALE) is a method for strain engineering to provide tolerance against a stress condition [11, 12]. During ALE, cells are repeatedly subcultured for prolonged periods under stress conditions leading to a reduced growth rate. This allows the selection of mutants with improved growth phenotypes under the stress environment [13]. Compared to random mutagenesis approaches using mutagens such as chemicals and ultraviolet light, ALE with sequential serial passages is a relatively easy method for identifying key mutations related to the improvement because of its low mutation frequency [14]. The productivity of the target compound can be enhanced by introducing the identified mutations that improve the tolerance of the strain into an engineered producer. Because a complete genome sequence of *Synechocystis* sp. PCC 6803 has already been determined [15], mutation positions are easily identified by genome resequencing analysis, which aligns genomic reads of the evolved strain to a reference genome [16]. The mutations for improving acid stress tolerance in *Synechocystis* sp. PCC 6803 were obtained through ALE experiments under low pH conditions and by whole genome resequencing analysis [17].

It has been reported that cells acquiring a stress tolerance exhibit a tolerance to the same type of stress [18, 19]. In the present study, we aimed to obtain a tolerant strain of *Synechocystis* sp. PCC 6803 against various types of alcohols using ALE with medium containing isobutanol, which has strong toxicity. Mutations of evolved strains were identified by whole genome resequencing and were introduced into the genome of the parental strain to evaluate the contribution for alcohol tolerance by a reverse genetics approach. We demonstrated that the acquired resistance ability of the evolved tolerant strain enhances ethanol production.

## Results

### Adaptive laboratory evolution of *Synechocystis* sp. PCC 6803 under isobutanol stress conditions

Isobutanol-tolerant strains were obtained via long-term passage culture experiments. Prior to passage cultures, *Synechocystis* sp. PCC 6803 was cultured under different isobutanol concentrations (0, 1, 2, 3, and 5 g/L) to determine the appropriate concentration for ALE. The OD_730_ at 72 h decreased by one-half in medium containing 2 g/L isobutanol compared to samples under no isobutanol condition (Additional file 1: Fig. S1). Passage cultures were performed using medium containing 2 g/L isobutanol. Because various evolved strains with different mutations can be obtained in ALE experiments, four independent passage cultures were performed. The relationship between the specific growth rate and accumulated culture time during ALE experiments are shown in Additional file 1: Fig. S2. The specific growth rates obviously increased until 500 h in the all four cultures. Although cell aggregation was observed until 300 h, cells eventually separated. Because no increase in the specific growth rate was observed after 1000 h, the evolved cells at 1824 h were stored at − 80 °C. Four strains, named T(1), T(2), T(3), and T(4), were isolated from the stored cultures of Nos. 1, 2, 3, and 4, respectively.

**Fig. 1:**
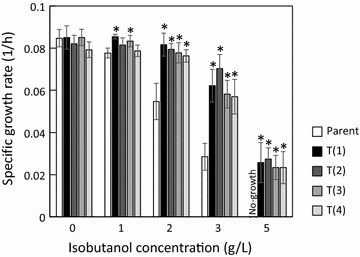
Specific growth rates of the parental and the evolved strains under different concentrations of isobutanol. The asterisks indicate significant differences between the parental and the evolved strains (t test, P < 0.05). The specific growth rate was calculated from an OD_730_ of 0 and 48 h of each batch culture. Error bars represent standard deviations of three replicates

**Fig. 2:**
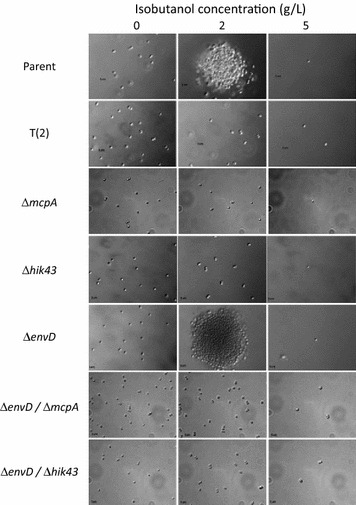
Cell flocculation of parental, evolved, and knockout strains at different isobutanol concentrations (0, 2, and 5 g/L)

To evaluate the isobutanol tolerance of the four evolved strains obtained from the ALE experiments, the parental and the evolved strains were cultured at different isobutanol concentrations (0, 1, 2, 3, and 5 g/L). The specific growth rate of the evolved strains increased compared to that of the parental strain with more than 2 g/L isobutanol (Fig. 1). Furthermore, the evolved strain grew in the presence of 5 g/L isobutanol which completely inhibited the growth of the parental strain. Although cell aggregation was observed in the parental strain, this was not observed in the evolved strains exposed to 2 g/L isobutanol (Fig. 2 and Additional file 1: Fig. S3).

### Whole genome resequencing of the evolved strains

**Fig. 3:**
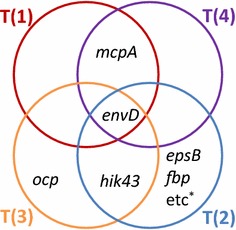
Identified mutations of the evolved strains by genome resequencing. These mutations were detected in 80% of the corresponding reads, and were confirmed by Sanger sequencing. *The T(2) strain possesses eight other mutations described in Additional file 1: Table S1

To investigate the mutations within the evolved strains, whole genome resequencing was performed using a MiSeq sequencer through paired-end sequencing with a read length of 250 bp. Coverage depth was approximately 100–200 reads. Identified mutations of the evolved strains are summarized as a Venn diagram in Fig. 3. The numbers of mutations in T(1), T(2), T(3), and T(4) strains were 2, 12, 2, and 2, respectively. T(1) and T(4) strains possessed mutations in *mcpA*, whereas T(2) and T(3) strains possessed mutations in *hik43*. All four evolved strains possessed mutations in *envD*. No mutation was identified in non-cording regions. Detailed information about the mutations is summarized in Fig. 3 and Table 1. Because the T(2) strain possessed a stop codon mutation in *mutS*, which encodes DNA mismatch repair protein, a higher number of mutations were accumulated during ALE than that in other evolved strains. It has been reported that *mcpA* and *hik43* are involved in the biogenesis of pili for generating motile force [20], and *envD* is involved in the transport of toxin [21]. The genome resequencing results suggest that these common mutations of *mcpA*, *hik43*, and *envD* in the evolved strains could be related to the observed enhanced isobutanol tolerance.

**Table 1:** Details about the mutations of evolved strains

Strain	Type^a^	Position	Reference	Mutation	Gene	Type	Mutation ratio (%)^b^	Function
T(1)	Del	492349	ATCCTA	-T----	slr1044 (*mcpA*)	Frameshift, stop codon	95	Methyl-accepting chemotaxis protein
	Ins	2364114	CCG	CCGA	slr0369 (*envD*)	Frameshift, stop codon	94	RND multidrug efflux transporter
T(2)	Ins	345787	TGG	TGGG	sll0923 (*epsB*)	Frameshift, stop codon	92	Unknown protein
	Ins	389860	CCC	CCCC	sll1025	Frameshift, stop codon	86	Hypothetical protein
	Ins	549361	CGG	CGGG	sll1636 (*fbp*)	Frameshift, stop codon	84	Ferripyochelin-binding protein
	SNP	755131	CTG	TTG	sll1924 (*sycrp2*)	Leu→Leu	99	cAMP receptor protein
	Ins	1041211	GGG	GGGG	slr1233 (*frdA*)	Frameshift, stop codon	94	Succinate dehydrogenase flavoprotein subunit
	Del	2270044	GGG	-GG	slr0322 (*hik43*)	Frameshift, stop codon	93	Two-component hybrid sensor and regulator
	SNP	2279149	CAG	GAG	slr0326	Gln→Glu	97	Hypothetical protein
	SNP	2364333	GTT	GGT	*envD*	Val→Gly	100	RND multidrug efflux transporter
	Del	2585782	CCC	-CC	sll0058 (*dnaK*)	Frameshift, stop codon	93	Heat-shock protein 70, molecular chaperone
	Del	3430453	CTT	C-T	slr0756 (*kaiA*)	Frameshift, stop codon	95	Circadian clock protein KaiA homolog
	Ins	1372331	CAA	CAAC	sll1165 (*mutS*)	Frameshift, stop codon	80	DNA mismatch repair protein MutS
T(3)	Del	1766850	ACCGCCCTTCCAACG	AC------------G	slr1963 (*ocp*)	Del (Ala, Leu, Pro)	88	Water-soluble carotenoid protein
	SNP	2271565	TGG	TGA	*hik43*	Stop codon	98	Two-component hybrid sensor and regulator
	Del	2366401	TCGGGGCGA	---------	*envD*	Del (Ser, Gly, Arg)	88	RND multidrug efflux transporter
T(4)	Ins	492262	TAT	TATT	*mcpA*	Frameshift, stop codon	95	Methyl-accepting chemotaxis protein
	SNP	2366659	GTT	TTT	*envD*	Val→Phe	99	RND multidrug efflux transporter

^a^Del, Ins, and SNP indicate an deletion, insertion, and single nucleotide polymorphism, respectively

^b^Mutation ratios indicates

Because the mutations of *mcpA* in T(1) and T(4) strains lead to a frame shift and a premature stop codon, the resulting putative truncated products are 75 and 30 amino acid residues, respectively, out of 869 amino acid residues. These truncated products must have no function due to their short length. The mutation of *hik43* in T(2) also lead to a frame shift and a premature stop codon. As the result, the putative truncated product is only 252 amino acid residues out of 1095 amino acid residues. The mutation of *envD* in T(1) also lead to a frame shift and a premature stop codon. As the result, the putative truncated product is only 158 amino acid residues out of 1083 amino acid residues. On the other hands, the putative products of EnvD in T(2), T(3), and T(4) strains had only few amino acid substitutions.

### Effect of mutations on isobutanol tolerance

To evaluate the effect of the mutations in *mcpA*, *hik43*, and *envD* of the evolved strains on isobutanol tolerance, the mutations and corresponding gene deletions were introduced into the genome of the parental strain.

*mcpA* encodes a methyl-accepting chemotaxis protein. No growth recovery was observed by introducing the *mpcA* deletion in the presence of 2 g/L isobutanol (Additional file 1: Fig. S4). Cell aggregation, which was observed in the parental strain, was dispersed in the ∆*mcpA* strain (Fig. 2 and Additional file 1: Fig. S5). This indicates that *mcpA* contributes to cell aggregation under the isobutanol stress condition. The strain did not grow in the presence of 5 g/L isobutanol, as well as its parental strain. The mcpA-R1 and mcpA-R4 strains were constructed by introducing *mcpA* mutations derived from the T(1) and T(4) strains, respectively, into the ∆*mcpA* strain. Because the mcpA-R1 and mcpA-R4 strains showed a similar phenotype as the ∆*mcpA* strain, the *mcpA* mutations were considered loss-of-function mutations.

**Fig. 4:**
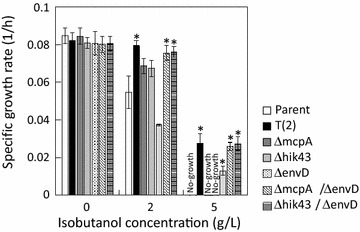
Specific growth rates of the ∆*mcpA*/∆*envD* and ∆*hik43*/∆*envD* strains under different concentrations of isobutanol. The asterisks indicate significant differences between the parental and the other strains (t test, P < 0.05). The specific growth rate was calculated from an OD_730_ of 0 and 48 h of each batch culture. Error bars represent standard deviations of three replicates

*hik43* encodes a histidine kinase, which is a member of the two-component hybrid sensor and regulator. Because stop codon mutations occurred in *hik43* of the T(2) and T(3) strains (Table 1), these mutations were also considered loss-of-function mutations. The ∆*hik43* strain showed the same growth phenotype as the *mcpA* mutants in the presence of isobutanol (Fig. 4). Cell aggregation was also dispersed in the ∆*hik43* strain in the presence of 2 g/L isobutanol (Fig. 2), indicating that *hik43* also contributes to cell aggregation under alcohol stress.

*envD* encodes the resistance nodulation division multidrug efflux transporter protein. Although the specific growth rate of the ∆*envD* strain decreased compared to the parental strain, in the presence of 2 g/L isobutanol, the ∆*envD* strain grew in the presence of 5 g/L isobutanol, which completely inhibits the growth of the parental strain (Fig. 4 and Additional file 1: Fig. S6). The specific growth rate of the ∆*envD* strain was lower than that of the evolved strain. The envD-R1, envD-R2, envD-R3, and envD-R4 strains were constructed by introducing *envD* mutations derived from the T(1), T(2), T(3), and T(4) strains, respectively, into the ∆*envD* strain. Cell aggregation was observed in all *envD* mutants in the presence of 2 g/L isobutanol as well as the parental strain (Fig. 2 and Additional file 1: Fig. S7). Because the envD-R1, envD-R2, envD-R3, and envD-R4 strains showed a similar phenotype to the ∆*envD* strain (Additional file 1: Fig. S6), the *envD* mutations were also considered loss-of-function mutations.

The specific growth rates of the constructed strains with single mutations of *mcpA*, *hik43*, and *envD* were lower than that of the evolved strains obtained from ALE experiments (Fig. 4). Because all four evolved strains possess combinatorial mutations of *mcpA* and *envD*, or that of *hik43* and *envD* (Fig. 3), it was assumed that the evolved strains acquired the isobutanol tolerance ability through the synergistic effect of these mutations. Since these *mcpA*, *hik43*, and *envD* mutations are considered loss-of-function mutations, the isobutanol tolerance of double deletion mutants (∆*mcpA*/∆*envD* and ∆*hik43*/∆*envD* strains) were evaluated. As expected, the ∆*mcpA*/∆*envD* and ∆*hik43*/∆*envD* strains showed identical growth with the evolved strain under 0, 2, and 5 g/L isobutanol concentrations (Fig. 4). Cell aggregation dispersed in ∆*mcpA*/∆*envD* and ∆*hik43*/∆*envD* strains in the presence of 2 g/L isobutanol (Fig. 2). These findings suggest that the evolved strains acquire isobutanol tolerance through the loss of function of *mcpA* and *envD*, or that of *hik43* and *envD*.

### Evaluation of stress tolerance against other alcohols of the evolved strains

**Fig. 5:**
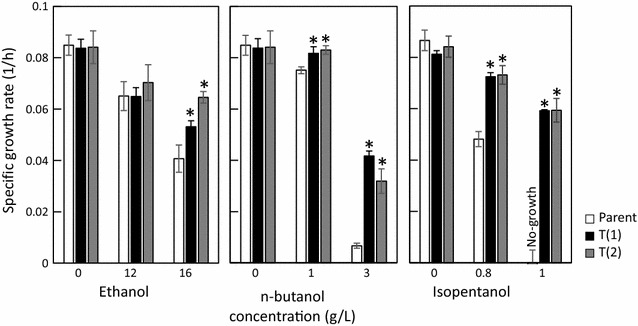
Tolerances of the parental and the evolved strains against ethanol, n-butanol, and isopentanol. The asterisks indicate significant differences between the parental and the evolved strains (t test, P < 0.05). The specific growth rate was calculated from the OD_730_ of 0 and 48 h of each batch culture. Error bars represent standard deviations of three replicates

Cells acquiring stress tolerance exhibit a tolerance to the same type of stress [18, 19]. Tolerance of the evolved strains against other alcohols such as ethanol, *n*-butanol, and isopentanol were evaluated (Fig. 5). The mutations important for alcohol tolerance were combinatorial malfunctions of *envD* and *mcpA*, or that of *envD* and *hik43*. Since both T(1) and T(4) strains have functional deficits in *mcpA* and *envD*, and both T(2) and T(4) strains have functional deficits in *hik43* and *envD* (Fig. 3), alcohol tolerances were evaluated for representative T(1) and T(2) strains. The specific growth rate of the evolved strains increased compared to the parental strain in the presence of 16 g/L ethanol, which contains less carbon atoms than isobutanol. In the presence of 1 and 3 g/L *n*-butanol, which has the same number of carbon atoms of isobutanol with a different main chain, the specific growth rate of the evolved strains increased compared to the parental strain. In the presence of 0.8 and 1 g/L isopentanol, which contains more carbon atoms than isobutanol with the same main chain, the specific growth rate of the evolved strains also increased compared to the parental strain (Fig. 5). These findings suggest that the evolved strains obtained by ALE under isobutanol stress exhibited tolerance against isobutanol as well as ethanol, *n*-butanol, and isopentanol.

### Ethanol production using the evolved strain

**Fig. 6:**
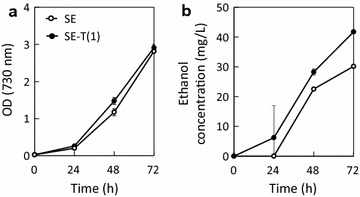
Culture profiles of SE and SE-T(1) strains. a cell growth and b ethanol concentration

The evolved strains showed tolerance against not only isobutanol but also ethanol (Fig. 5). The effect of alcohol tolerance on ethanol production was investigated. Ethanol-producing strains of the parental and T(1) strains, called SE and SE-T(1) strains, were constructed by introducing heterologous genes for *pdc* and *adhII* from *Zymomonas mobilis*. Culture profiles of SE and SE-T(1) strains are shown in Fig. 6. Although the specific growth rate of the SE-T(1) strain was slightly lower than that of the SE strain, enhanced ethanol production was observed in the SE-T(1) strain. The ethanol productivity at 72 h of SE and SE-T(1) strains were 10.4 ± 0.4 and 14.8 ± 0. 2 mg L^−1^ OD^−1^, respectively.

## Discussion

In this study, evolved strains of *Synechocystis* sp. PCC 6803 were obtained using the ALE method in medium containing 2 g/L isobutanol, to engineer a strain with strong tolerance against various alcohols (Fig. 5). Genome resequencing analysis demonstrated that the evolved strains possess mutations in *mcpA* and *envD*, or *hik43* and *envD* (Fig. 3). Reverse genetics approaches revealed that the mutations of *mcpA*, *hik43*, and *envD* were loss-of-function mutations, and the evolved strains acquired resistance by combinatorial malfunctions of *envD* and *mcpA*, or that of *envD* and *hik43* (Fig. 4).

In the ∆*mcpA* and ∆*hik43* strains, since no cell aggregation was observed in the presence of 2 g/L isobutanol, *mcpA* and *hik43* genes are likely related to cell flocculation. It has been reported that deletion of *mcpA* or *hik43* causes a loss of thick pili, which is essential for phototactic motility in *Synechocystis* sp. PCC 6803 [20]. Although the thick pili play a role in uptake of extracellular DNA in nature, these pili draw not only extracellular DNA but also the surrounding cells under isobutanol-induced stress. Cell aggregation of *Synechocystis* sp. PCC 6803 was also observed under *n*-butanol stress [22]. Transcriptome analysis demonstrated the up-regulation of genes involved in chemotaxis [6]. The relationship between cell flocculation size and ethanol tolerance has been investigated in yeast. Yeast has improved ethanol tolerance as flocculation size increases. On the other hand, in this study, alcohol tolerance was improved by making free of flocculation, so the tolerance was acquired by a mechanism different from yeast [23, 24].

The amino acid sequence of a protein encoding *envD* is similar to that of AcrB and AcrD of *E. coli*. Pairwise alignments show that both proteins share 38% amino acid sequence identity. Amino acid sequence similarities of the EnvD to the *E. coli* AcrB and AcrD are 58 and 59%, respectively. *E. coli* AcrB and AcrF have been categorized into a same orthologous protein cluster of EnvD in CyanoClust for the protein homology database featuring cyanobacteria and plastids [25]. The AcrB is a component of the AcrAB-TolC transporter, which can export a broad variety of compounds with little chemical similarity [26]. It has been reported that the deletion of AcrAB-TolC contributed to enhance tolerance against solvents such as styrene, 1-octanol, and isobutanol [27]. Isobutanol stress causes leakage of quinone, an electron acceptor in the *E. coli* respiratory chain, owing to membrane damage [28]. Because AcrAB-TolC can also export quinone compounds [29] and is highly induced by isobutanol stress [28], the deletion of AcrAB-TolC increased isobutanol tolerance with reducing the quinone [18]. Although, to our knowledge, there are no studies on the molecular function of EnvD in *Synechocystis* sp. PCC 6803, its amino acid sequence similarity with AcrB would also be expected to involve quinone export. The *envD* deletion strain could acquire isobutanol tolerance with alleviation of quinone sink under the isobutanol stress condition. Despite the fact that the *envD* deletion makes it possible for the strain to grow in the presence of 5 g/L isobutanol, the specific growth rate of the *envD* deletion strain decreased compared to the parental strain in the presence of 2 g/L isobutanol. Because deletion of membrane and membrane-associated proteins, such as transporters, would affect membrane lipids [27], the *envD* deletion negatively affected the cell growth indirectly in the presence of 2 g/L isobutanol.

Since the ALE experiments were conducted using 2 g/L isobutanol, the mutants of *envD* should not be selected because of its negative effect for the tolerance to this isobutanol level. Synergistic effects for isobutanol tolerance induced by the combinatorial gene deletions of *envD* and *mcpA*, or that of *envD* and *hik43* are shown in Fig. 4. The findings indicate that first the mutations in *mcpA* or *hik43* were introduced, followed by the mutations in *envD*, since non-aggregated cells by *mcpA* or *hik43* mutation are easy to subculture in the passaged cultures. Although the mechanisms of the synergistic effect for isobutanol tolerance by these two genes are still unclear, photon supply to each cell increases through interference with cell aggregation. Additionally, the metabolic activities could be enhanced through alleviation of quinone sink with *envD* mutations. It is difficult to rationally predict such synergistic effects for tolerance by multiple gene deletions. The ALE method has become a powerful tool for strain improvement to obtain stress tolerance.

The expression of small heat-shock protein HspA (sll1514) and hypothetical protein CccS (slr1617) has improved 1-butanol tolerance [6]. Furthermore, it is known that a response regulator (Slr1037) is involved in butanol tolerance of *Synechocystis* sp. PCC 6803 [7], and co-overexpression of slr1037 and sll0039 improves butanol tolerance by 133% [9]. An overexpression of SigB is also known to improve 1-butanol tolerance in *Synechocystis* sp. PCC 6803 [10]. By understanding the mechanism of the alcohol tolerance acquisition by the combinatorial malfunctions of *envD* and *mcpA*, or that of *envD* and *hik43*, it may help to further enhance the alcohol tolerance in coordination with these previous findings.

The SE-T(2) strain, in which ethanol-producing genes were introduced, shows enhanced ethanol production compared to the control strain. This indicates that the ethanol tolerance ability is effective for enhancing the ethanol production rate. Since ethanol affected the growth of the parental strain at concentrations of up to 16 g/L (Fig. 5), a higher ethanol concentration was required to inhibit growth of the SE-T(2) strain (28.3 mg/L at 48 h). If ethanol excretion is a rate-limiting step, the ethanol concentration in the cells becomes much higher than that of the extracellular culture broth. Therefore, the ethanol-producing cells would be exposed to a stress condition in which the concentration of ethanol is higher than the produced ethanol concentration. Furthermore, the transporter malfunction may indirectly contribute to ethanol production. Because the mechanism is unknown, detailed metabolic analysis is necessary to clarify this.

In this study, we chose ethanol, which has been widely studied in cyanobacterial bio-production as a target alcohol. Since the evolved strains showed tolerance abilities against various alcohols (Fig. 5), the strains have the potential to enhance the production rate of a wide variety of alcohols. Since the mutation for enhancing alcohol tolerance was obtained by ALE experiments using isobutanol, it is expected to contribute in isobutanol productions, especially. Isobutanol showing more strong toxicity may clearly demonstrate the effect of the mutations.

## Conclusions

Alcohol stress tolerant strains of *Synechocystis* sp. PCC 6803 were obtained via the ALE method using medium containing isobutanol, which induces strong toxicity. The evolved strains showed increased growth rates in more than 2 g/L isobutanol, and could grow in the presence of 5 g/L isobutanol, which completely inhibits the growth of the parental strain. Genome resequencing and reverse genetics analyses revealed that the evolved strains acquired their resistive capacity owing to combinatorial malfunctions of *envD* and *mcpA*, or that of *envD* and *hik43*. Furthermore, the evolved strains also demonstrated tolerance against a wide variety of alcohols, such as ethanol, *n*-butanol, and isopentanol. The ethanol productivity successfully increased to 142% of the control strain by introducing an ethanol-producing pathway into the evolved strain.

## Methods

### Strain and culture condition

The *Synechocystis* sp. PCC 6803 glucose-tolerant strain served as the parent strain. All strains used in this study are listed in Table 1. Cultures were performed autotrophically using 100-mL Erlenmeyer flasks with 20 mL BG11 medium [30] at 34 °C, with rotary agitation at 150 rpm (BR-43FL shaker; TAITEC Co., Ltd., Japan) under continuous illumination (40 μmol m^−2^ s^−1^) by white light-emitting diodes (LC-LED450 W, TAITEC Co., Ltd., Japan). The BG11 medium was supplemented with 50 mM NaHCO_3_ and the required antibiotics.

### Analytical methods

Cell growth was measured as optical density at 730 nm (OD_730_) using a spectrophotometer (UVmini-1240, Shimadzu, Kyoto, Japan). The ethanol concentration of the culture was measured by gas chromatography with a hydrogen flame ionization detector (7890A; Agilent Technologies, CA, USA) and a Stabiliwax column (0.32 mm internal diameter, 60 m length, and 1 μm thickness; Restek, PA, USA). The analytical conditions are described elsewhere [31]. A light optical microscope (Imager M2, Carl Zeiss, Jena, Germany) with AxioVision Release 4.8 was used to examine the flocculation of the cells.

### Tolerance test of alcohols

Cells were inoculated at OD_730_ of 0.05 for pre-culture, and then incubated in the above conditions. After 3 days, the pre-cultivated cells were harvested by centrifugation, and then inoculated into the fresh BG11 medium at OD_730_ of 0.03. To test the alcohol tolerance, an appropriate amount of 99% (w/w) isobutanol, 99.5% (w/w) ethanol, 99% (w/w) *n*-butanol, or 98% (w/w) isopentanol was supplemented into the medium. A silicon cap was used in the culture to prevent evaporation of alcohols from the culture broth. The specific growth rate was calculated from OD_730_ during the exponential growth phase (0–48 h).

### Adaptive laboratory evolution

Sequential serial passages were performed using the parental strain and BG11 medium with 2 g/L isobutanol under the previously mentioned culture conditions. Cells were inoculated into fresh medium every 3.5 days at OD_730_ of 0.03 to maintain the growth phase in four parallel cultures. A silicon cap was used to prevent the evaporation of isobutanol. The specific growth rate was calculated from OD_730_ up to 0–48 h for each culture. After the final cultivation, cells were stored at − 80 °C in a 15% glycerol solution. The freeze stock was streaked on a BG11 plate for single colony isolation. The obtained single colonies were inoculated in BG11 medium, and cultured with 2 g/L isobutanol. The strains were stored at − 80 °C in a 15% glycerol solution, and labeled as T(1), T(2), T(3), and T(4).

### Whole genome sequencing

The genomic DNAs of the parental and its evolved strains were extracted using a genome extraction kit (Qiagen, CA, USA). The DNA samples were sequenced using a MiSeq sequencer (Illumina) with the MiSeq Reagent kit v2 (Illumina) generating 150 bp paired-end reads. The sequenced reads were mapped to the genome sequence of the *Synechocystis* sp. PCC 6803 GT-I strain (NCBI Reference Sequence No.: NC_017038.1) [16] using the Bowtie 2 software ver. 2.2.3 [32]. The SNPs of the parental strain to the reference genome were identified using SAMtools ver 1.0. The SNPs of the evolved strains were also checked using the Integrative Genomics Viewer [33]. The SNPs of the evolved strains identified by whole genome sequencing were confirmed by Sanger sequencing (Eurofins) using DNA fragments, including the mutation site amplified by PCR with the primer pair listed in Additional file 1: Table S1.

### Genomic manipulations

Strains used in this study are listed in Table 2. Single gene deletion strains of ∆*mcpA*, ∆*hik43*, and ∆*envD* were constructed using *Synechocystis* sp. PCC 6803 glucose-tolerant strain as the parental strain by replacing each corresponding gene with a kanamycin-resistance gene. To construct strains in which mutations were identified after ALE, the kanamycin-resistance gene in the corresponding gene deletion strain was replaced with the mutated gene and a streptomycin-resistance gene. Double gene deletion strains of ∆*mcpA*/∆*envD* and ∆*hik43*/∆*envD* were constructed using the ∆*envD* strain by replacing *mcpA* or *hik43* with a streptomycin-resistance gene. Complete segregation of the target site by the antibiotic resistance gene was confirmed by the disparity in the sizes of the PCR products generated using the primers that bound outside the upstream and downstream regions of target site for homologous recombination. Detailed procedures for strain construction, including plasmid and primer information, are described in an additional document.

**Table 2:** Strains used in this study

Strains	Description	References
Parent	*Synechocystis* sp. PCC 6803 GT-I	[31]
T(1)	Obtained from ALE in No. 1	This work
T(2)	Obtained from ALE in No. 2	This work
T(3)	Obtained from ALE in No. 3	This work
T(4)	Obtained from ALE in No. 4	This work
∆*mcpA*	*mcpA*::Km^r^	This work
∆*hik43*	*hik43*::Km^r^	This work
∆*envD*	*envD*::Km^r^	This work
mcpA-R1	*mcpA*::*mcpA * T(1) Sm^r^	This work
mcpA-R4	*mcpA*::*mcpA * T(2) Sm^r^	This work
envD-R1	*envD*::*envD* T(1) Sm^r^	This work
envD-R2	*envD*::*envD* T(2) Sm^r^	This work
envD-R3	*envD*::*envD* T(3) Sm^r^	This work
envD-R4	*envD*::*envD* T(4) Sm^r^	This work
∆*envD*/∆*mcpA*	*envD*::Km^r^,* mcpA*::Sm^r^	This work
∆*envD*/∆*hik43*	*envD*::Km^r^,* hik43*::Sm^r^	This work
SE	*ndhB*::P_psbA2_ * pdc* * adhII* Am^r^	[31]
SE-T(1)	T(1)* ndhB*::P_psbA2_ * pdc* * adhII* Am^r^	This work

## Additional file

**Additional file 1.** Additional figures, table, and details procedures for strain construction.
