# Supplementary material for: Identification of alcohol stress tolerance genes of Synechocystis sp. PCC 6803 using adaptive laboratory evolution
Source: Biotechnol Biofuels. 2017 Dec 20;10:307. doi: 10.1186/s13068-017-0996-5 (PMC5738210; doi:10.1186/s13068-017-0996-5)
Supplement: Supplementary file 1 — Additional file 1. Additional figures, table, and details procedures for strain construction. [file 13068_2017_996_MOESM1_ESM.pdf]

Supplemental information

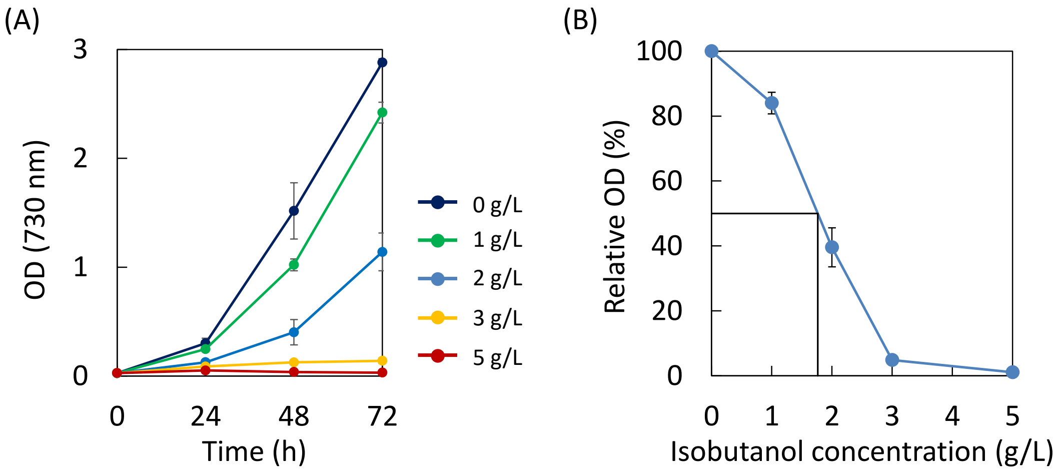

Fig. S1 Effect of isobutanol on the growth of *Synechocystis* sp. PCC 6803

(A) Time series of OD at 730 nm under different isobutanol concentrations (0, 1, 2, 3, and 5 g/L), (B) Relationship between cell growth and isobutanol concentration. Relative OD is normalized by the OD at 72 h with no isobutanol added. The OD value at 72 h of each condition is normalized by that of the no isobutanol condition.

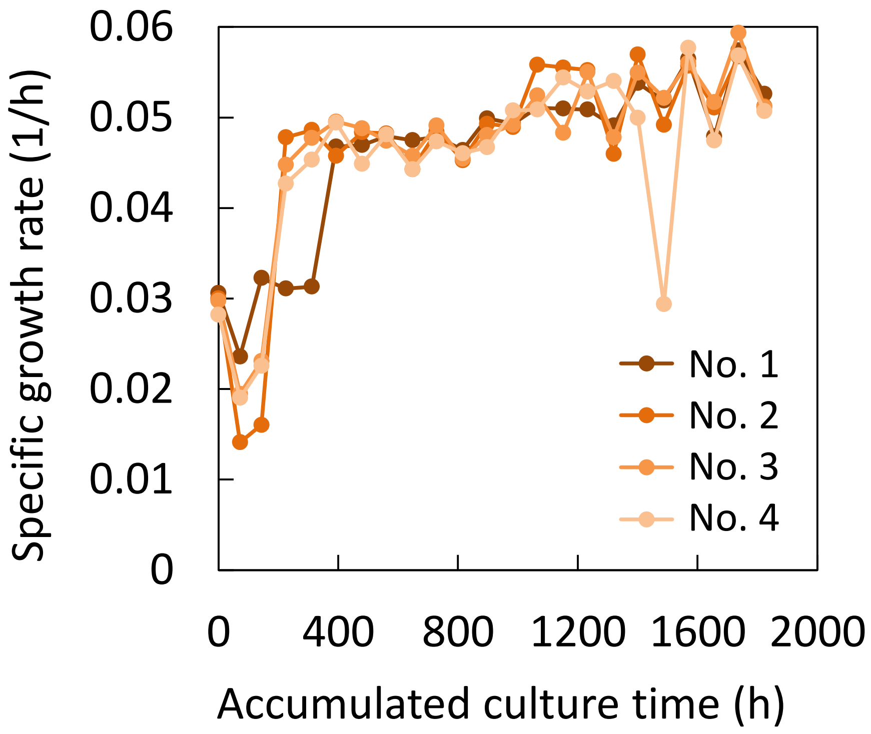

Fig. S2 Specific growth rates during ALE experiments in the presence of 2 g/L isobutanol

Four independent passage cultures, No. 1, No. 2, No. 3, and No. 4, were performed. The specific growth rate was calculated from the OD_{730} of 0 and 84 h of each batch culture.

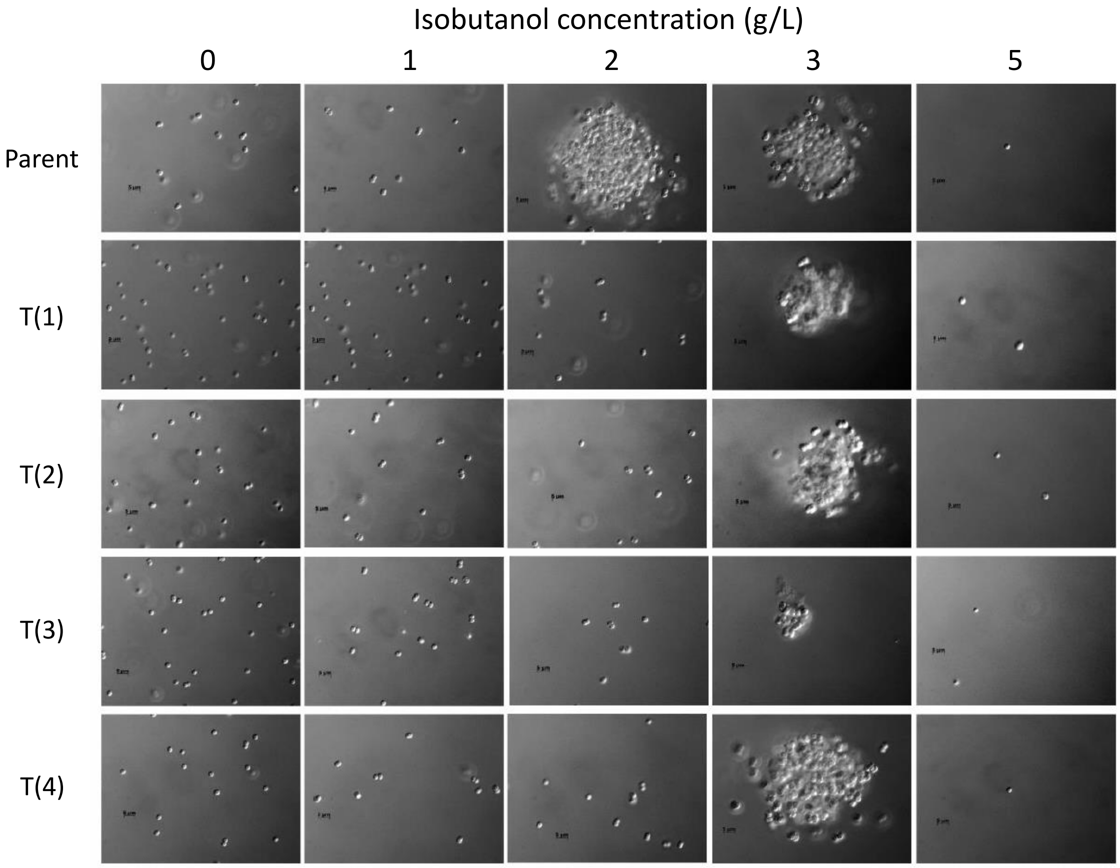

Fig. S3 Cell flocculation of the parental and evolved strains at different isobutanol concentrations (0, 1, 2, 3, and 5 g/L)

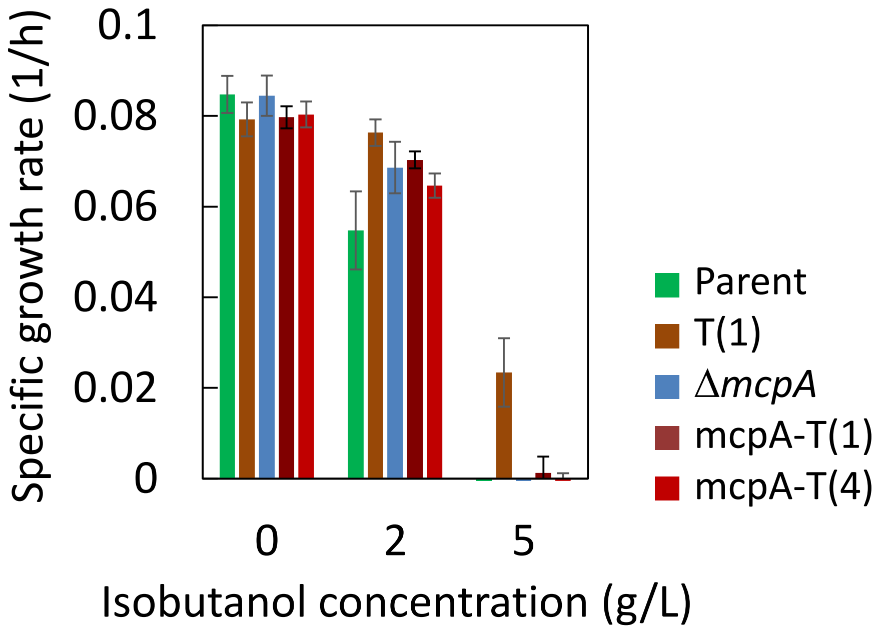

Fig. S4 Specific growth rate of Δ*mcpA* and *mcpA* mutant strains at different isobutanol concentrations (0, 2, and 5 g/L)

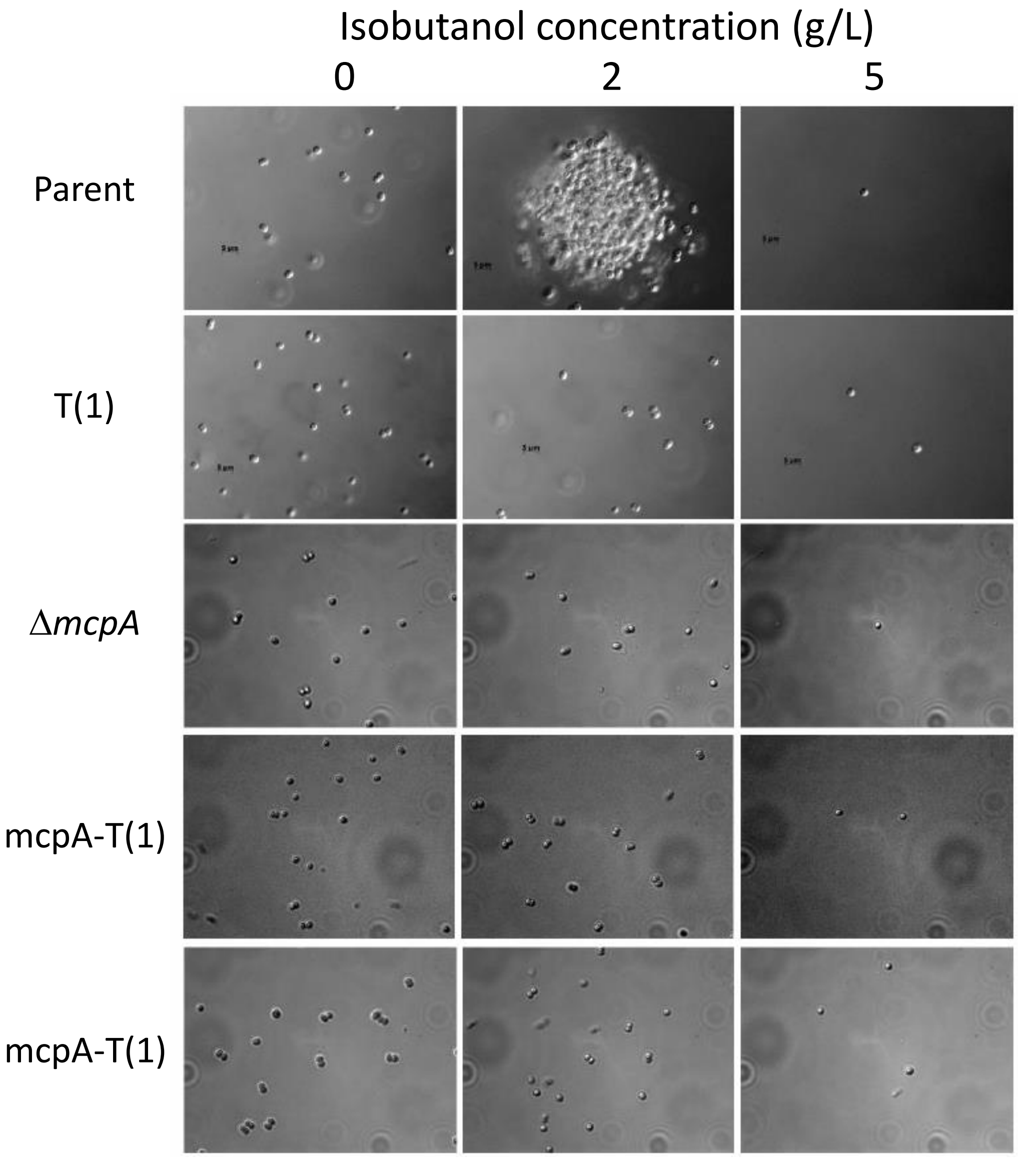

Fig. S5 Cell flocculation of Δ*mcpA* and *mcpA* mutant strains at different isobutanol concentrations (0, 2, and 5 g/L)

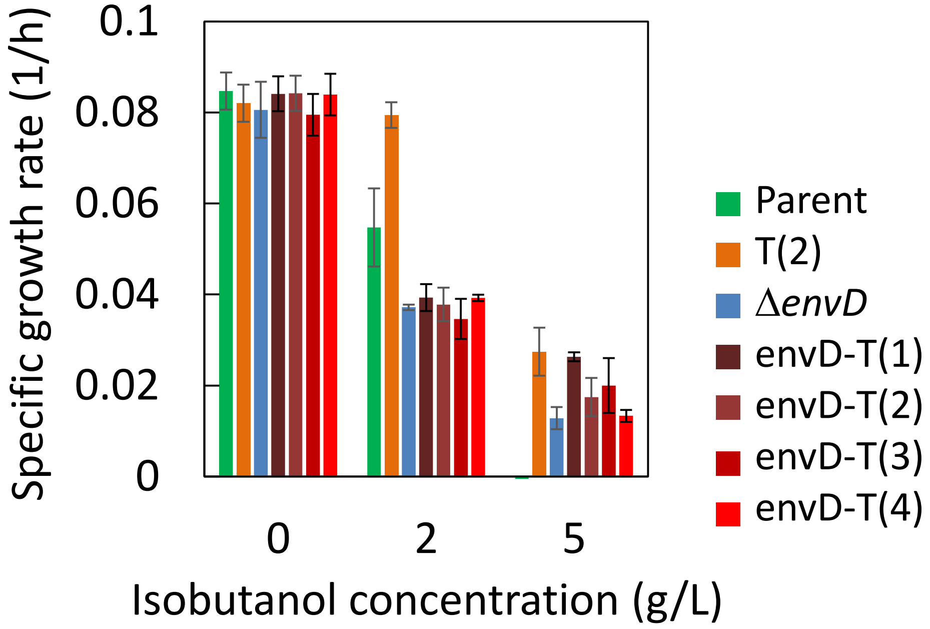

Fig. S6 Specific growth rate of Δ*envD* and *envD* mutant strains at different isobutanol concentrations (0, 2, and 5 g/L)

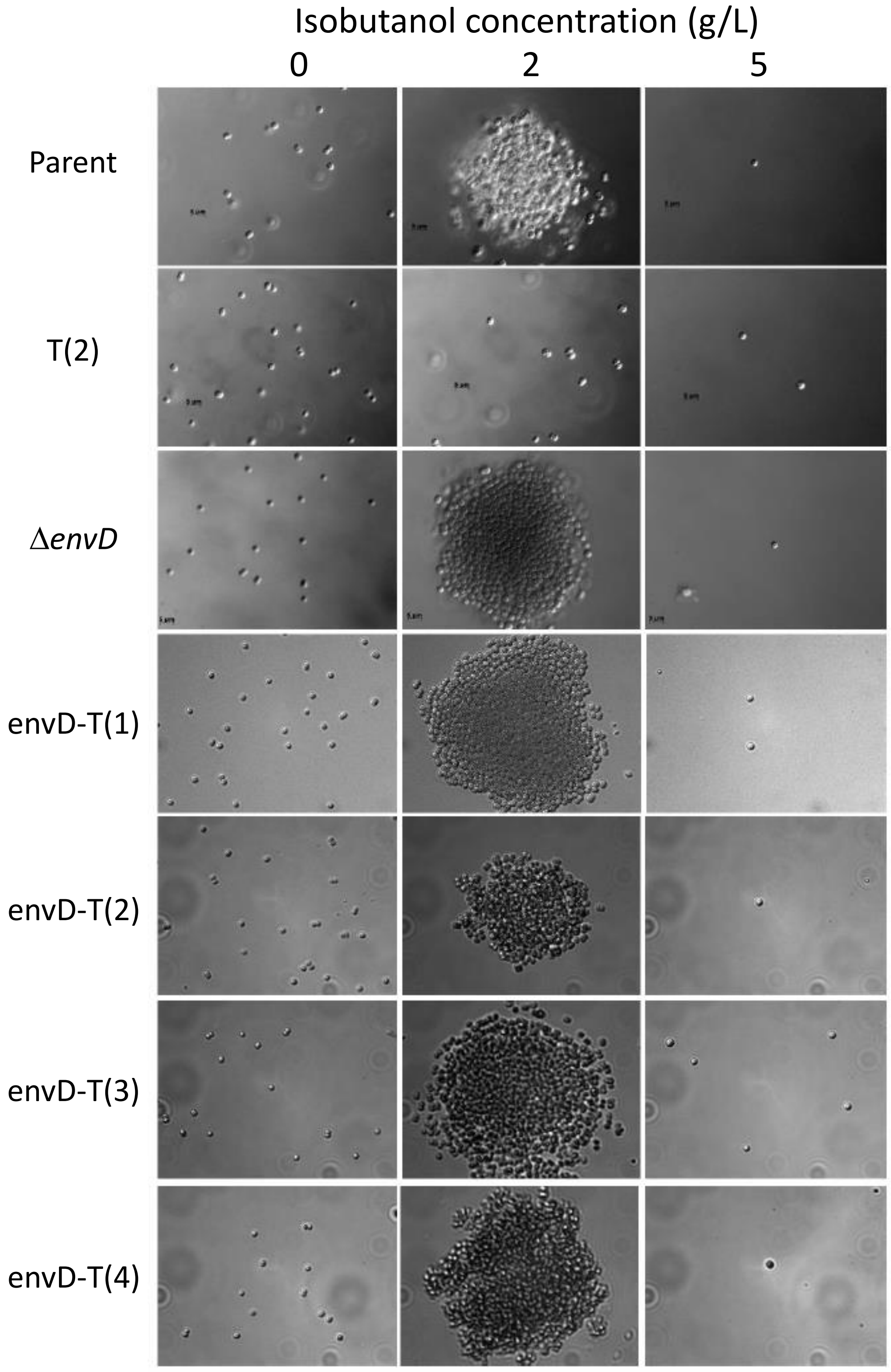

Fig. S7 Cell flocculation of ΔenvD and envD mutant strains at different isobutanol concentrations (0, 2, and 5 g/L)

Strain construction

All PCR primers for strain constructions are listed in Table S1.

Construction of the ΔmcpA strain

The *mcpA* deletion strain of *Synechocystis* sp. PCC 6803, named Δ*mcpA*, was constructed by replacing *mcpA* with a kanamycin resistance gene. The upstream and downstream regions of *mcpA* were amplified from the *Synechocystis* sp. PCC 6803 genome with PCR using the mcpA_up_F and mcpA_up_R_Km, and mcpA_down_F_Km and mcpA_down_R primer sets, respectively. The kanamycin resistance gene was amplified from a pHSG298 (Takara Bio, Siga, Japan) using the Km_F and Km_R primer set. KOD-plus-NEO (Toyobo, Japan), which was used for PCR. After purification using the FastGene Gel/PCR Extraction Kit (Fast Gene), these three PCR products were connected with overlap-extension PCR (OE-PCR) using the mcpA_up_F and mcpA_down_R primer set. The resulting PCR product was introduced into *Synechocystis* sp. PCC 6803. The transformants were screened on BG11 plates containing 20 μg/L of kanamycin. Complete segregation of *mcpA* by kanamycin resistance gene was confirmed by the disparity in the sizes of the PCR products generated using the mcpA_check_F and mcpA_check_R (check primer set), and the mcpA_int_F and mcpA_check_R (internal primer set).

The result of agarose gel electrophoresis is shown in Fig. S8. In the PCR using the internal primer set, an approximately 1000 bp PCR product was observed in the parental strain (lane 6), which was absent in the Δ*mcpA* strains (lanes 7 to 9). In the PCR with the check primer set, an approximately 4000 bp PCR product was observed in the parental strain (lane 1), whereas an approximately 2200 bp PCR product was observed in the Δ*mcpA* strains (lanes 2 to 4). These results are consistent with the fact that *mcpA* was replaced with a kanamycin resistance gene.

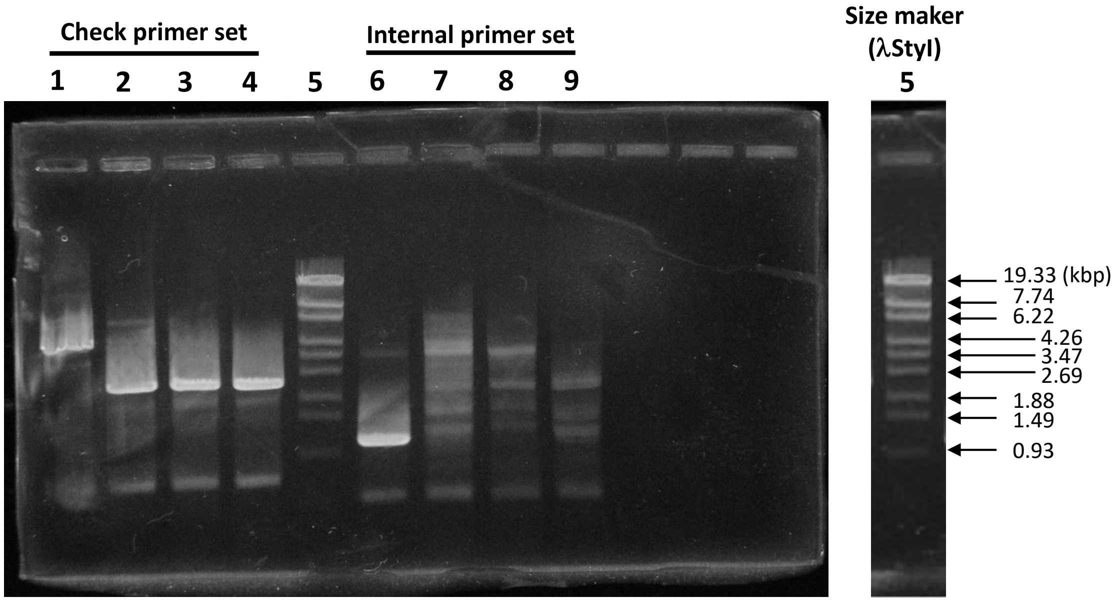

Fig. S8 Confirmation of Δ*mcpA* strain construction using PCR

Lanes 1 to 4 and 6 to 9 are PCR products amplified using the check and internal primer sets, respectively. Lane 5 is the DNA size maker of λStyI. Lanes 1 and 6 are PCR products from the parental strain. Lanes 2 to 4 and 7 to 9 are PCR products from the constructed Δ*mcpA* strains.

Construction of the Δhik43 strain

The *hik43* deletion strain (Δ*hik43*) was constructed by replacing *hik43* with a kanamycin resistance gene. The upstream and downstream regions of *hik43* were also amplified with PCR using the primer set of hik43_up_F and hik43_up_R_Km, and that of hik43_down_F_Km and hik43_down_R, respectively. The upstream region of *hik43*, the kanamycin resistance gene, and the downstream regions of *hik43* were connected with OE-PCR using the hik43_up_F and hik43_down_R primer set. The resulting PCR product was introduced into the parental strain. The transformants were screened on BG11 plates containing 20 μg/L of kanamycin. Complete segregation of *hik43* by kanamycin resistance gene was confirmed by the disparity in the sizes of the PCR products generated using the hik43_check_F and hik43_check_R (check primer set), and the hik43_int_F and hik43_check_R (internal primer set).

The result of agarose gel electrophoresis is shown in Fig. S9. In the PCR using the internal primer set, an approximately 1000 bp PCR product was observed in the parental strain (lane 1), which was absent in the Δ*hik43* strains (lanes 2 to 4). In the PCR with the check primer set, an approximately 5000 bp PCR product was observed in the parental strain (lane 6), whereas an approximately 2200 bp PCR product was

observed in the Δ*mcpA* strains (lanes 7 to 9). These results are consistent with the fact that *hik43* was replaced with a kanamycin resistance gene.

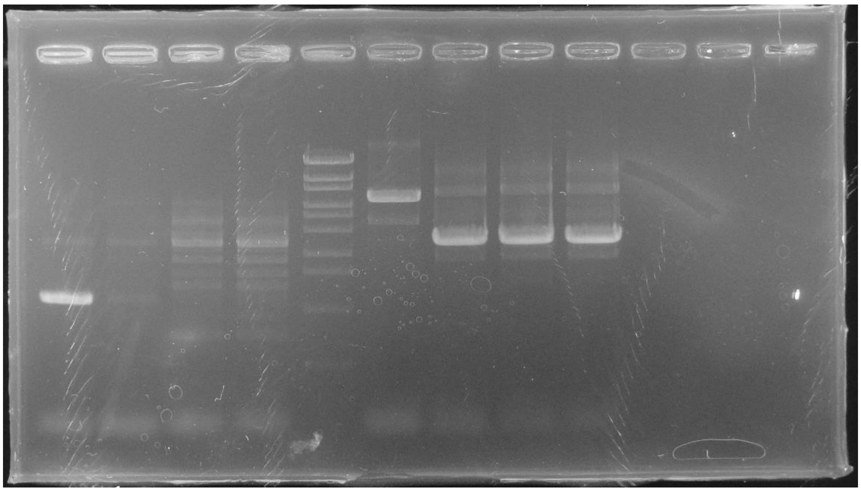

Fig. S9 Confirmation of Δ*hik43* strain construction using PCR
Lanes 1 to 4 and 6 to 9 are PCR products amplified using the internal and check primer sets, respectively. Lane 5 is the DNA size maker of λStyI (see Fig. S8 for size distribution). Lanes 1 and 6 are PCR products from the parental strain. Lanes 2 to 4 and 7 to 9 are PCR products from the constructed Δ*hik43* strains.

Construction of the ΔenvD strain

The *envD* deletion strain (Δ*envD*) was constructed by replacing *envD* with a kanamycin resistance gene. The upstream and downstream regions of *envD* were also amplified with PCR using the envD_up_F and envD_up_R_Km, and envD_down_F_Km and envD_down_R primer sets, respectively. The upstream region of *envD*, the kanamycin resistance gene, and the downstream regions of *envD* were connected with OE-PCR using envD_up_F and envD_down_R primer set. The resulting PCR product was introduced into the parental strain. The transformants were screened on BG11 plates containing 20 μg/L of kanamycin. Complete segregation of *envD* by the kanamycin resistance gene was confirmed by the disparity in the sizes of the PCR products generated using the envD_check_F and envD_check_R (check primer set) and envD_int_F and envD_check_R (internal primer set).

The result of agarose gel electrophoresis is shown in Fig. S10. In the PCR using the internal primer set, an approximately 1000 bp PCR product was observed in

the parental strain (lane 2), which was not present in the Δ*envD* strains (lanes 3 to 5). In the PCR using the check primer set, an approximately 5000 bp PCR product was observed in the parental strain (lane 6), whereas an approximately 2200 bp PCR product was observed in the Δ*mcpA* strains (lanes 7 to 9). These results are consistent with the fact that *envD* was replaced with a kanamycin resistance gene.

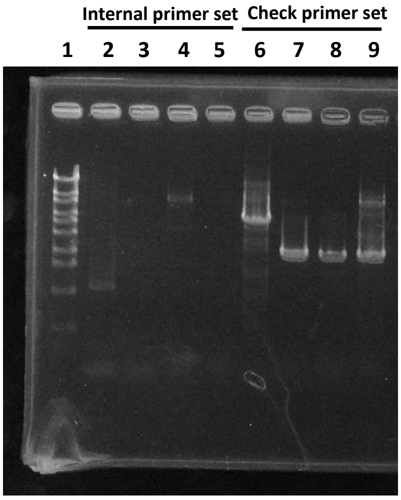

Fig. S10 Confirmation of Δ*envD* strain construction using PCR
Lanes 2 to 5 and 6 to 9 are PCR products amplified using the internal and check primer sets, respectively. Lanes 2 and 6 are PCR products from the parental strain. Lane 1 is the DNA size maker of λStyI (see Fig. S8 for size distribution). Lanes 3 to 5 and 7 to 9 are PCR products from the constructed Δ*envD* strains.

Introduction of mcpA mutations of the evolved strains into the ΔmcpA strain
The mcpA-R1 and mcpA-R4 strains were constructed by replacing the kanamycin resistance gene with mutated *mcpA* genes from evolved strains and a streptomycin resistance gene. Genomic DNAs of the evolved strains were extracted using a QIAquick Gel Extraction Kit (Qiagen). Mutated *mcpA* genes were amplified from the genomic DNA of the T(1) and T(4) strain by PCR using the mcpA_up_F and mcpA_down_R_BglII primer set. The PCR products were cloned into a pGEMt-Easy vector (Promega), and these sequences were confirmed. The down steam region of *mcpA* was also amplified by PCR using the mcpA_down_F_Sm and mcpA_down_R_BglII primer set. A streptomycin resistance gene was amplified from pCDFDuet-1 (Novagen) using the Sm_F_BglII and Sm_R primer set. The downstream region of *mcpA* and the streptomycin resistance gene were connected with OE-PCR using the Sm_F_BglII and mcpA_down_R_BglII primer set. The resulting PCR product

was digested by BglII, and was inserted into the BglII site of the engineered pGEMt vectors which has mutated a *mcpA* gene. The resulting plasmids were introduced into the ΔmcpA strain. The transformants were screened on BG11 plates containing 20 μg/L of streptomycin. Complete segregation of native *mcpA* by mutated *mcpA* and the streptomycin resistance gene was confirmed by the disparity in the sizes of the PCR products generated using the mcpA_check_F and mcpA_check_R (check primer set), and mcpA_int_F and mcpA_check_R (internal primer set).

The results of agarose gel electrophoresis are shown in Fig. S11. In the PCR using the check primer set, although approximately 4000 bp and 2200 bp PCR products were observed in the parental strain (lane 2) and ΔmcpA strain (lane 3), respectively, an approximately 6000 bp PCR product was observed in the mcpA-R1 and mcpA-R4 strains (lanes 4 to 6). In the PCR using the internal primer set, an approximately 1000 bp PCR product was observed in the ΔmcpA strain (lane 7), which was absent in the mcpA-R1 and mcpA-R4 strains (lanes 8 to 10). These results are consistent with the fact that the kanamycin resistance gene of the ΔmcpA strain was replaced with mutated *mcpA* and the streptomycin resistance gene.

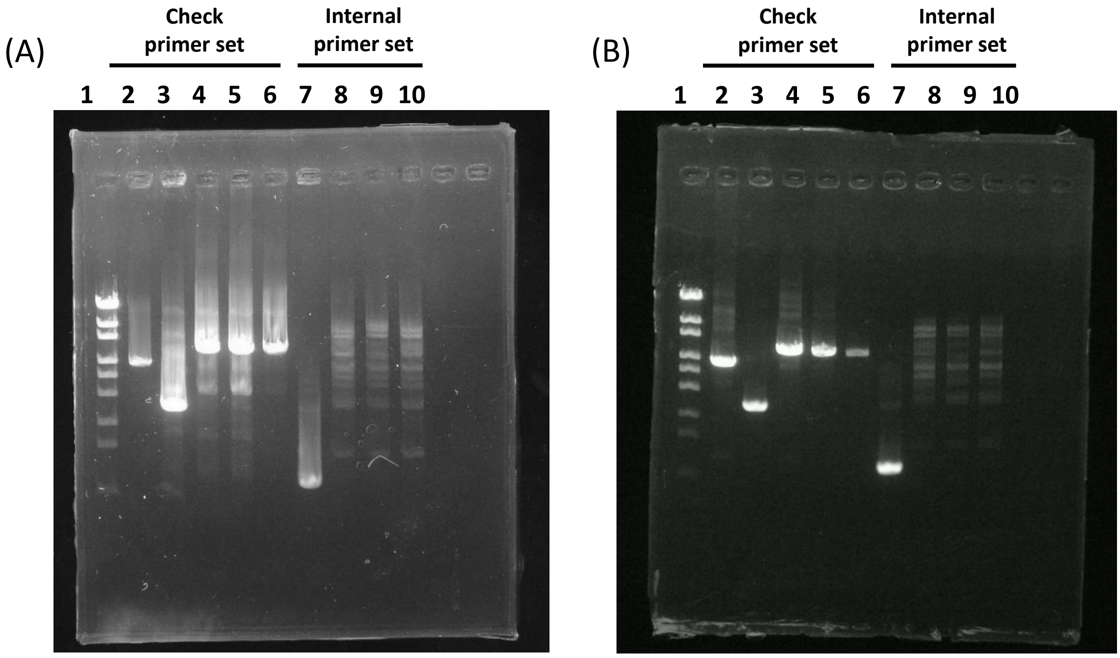

Fig. S11 Confirmation of strains with mcpA mutations using PCR

(A) mcpA-R1; (B) mcpA-R4

Lanes 2 to 6 and 7 to 10 are PCR products amplified using the check and internal primer sets, respectively. Lane 1 is the DNA size maker of λStyI (see Fig. S8 for size distribution). Lane 2 is PCR products from the parental strain. Lanes 3 and 7 are PCR

products from the ΔmcpA strain. Lanes 4 to 6 and 8 to 10 are PCR products from the constructed strains with *mcpA* mutations derived from the evolved strains.

*Introduction of envD mutations of the evolved strains into the ΔenvD strai*n

The envD-R1, envD-R2, envD-R3, and envD-R4 strains were constructed by replacing the kanamycin resistance gene of the ΔenvD strain with mutated *envD* genes from evolved strains and a streptomycin resistance gene. Mutated *envD* genes were amplified from the genomic DNA of each evolved strain by PCR using the envD_up_F and envD_down_R_BglII primer set. The PCR products were cloned into a pGEMt-Easy vector (Promega), and these sequences were confirmed. The down steam region of the *envD* was also amplified by PCR using the envD_down_F_Sm and envD_down_R_BglII primer set. The downstream region of *envD* and a streptomycin resistance gene (see in above section) were connected with OE-PCR using the Sm_F_BglII and envD_down_R_BglII primer set. The resulting PCR product was digested by BglII, and was inserted into the BglII site of the engineered pGEMt vectors, which has a mutated *envD* gene. The resulting plasmids were introduced into the ΔenvD strain. The transformants were screened on BG11 plates containing 20 μg/L of streptomycin. Complete segregation of native *envD* by mutated *envD* and streptomycin resistance gene was confirmed by the disparity in the sizes of the PCR products generated using the envD_check_F and envD_check_R (check primer set), and envD_int_F and envD_check_R (internal primer set).

The results of agarose gel electrophoresis are shown in Fig. S12. In the PCR using the check primer set, although approximately 4000 bp and 2200 bp of PCR products were observed in the parental (lane 2) and Δ*envD* (lane 3) strains, respectively, an approximately 6000 bp PCR product was observed in the envD-R1, envD-R2, envD-R3, and envD-R4 strains (lanes 4 to 6). In the PCR with the internal primer set, an approximately 1000 bp PCR product was observed in the Δ*envD* strain (lane 7), which was not observed in the envD-R1, envD-R2, envD-R3, and envD-R4 strains (lanes 8 to 10). These results are consistent with the fact that the kanamycin resistance gene of the ΔenvD strain was replaced with mutated *envD* and the streptomycin resistance gene.

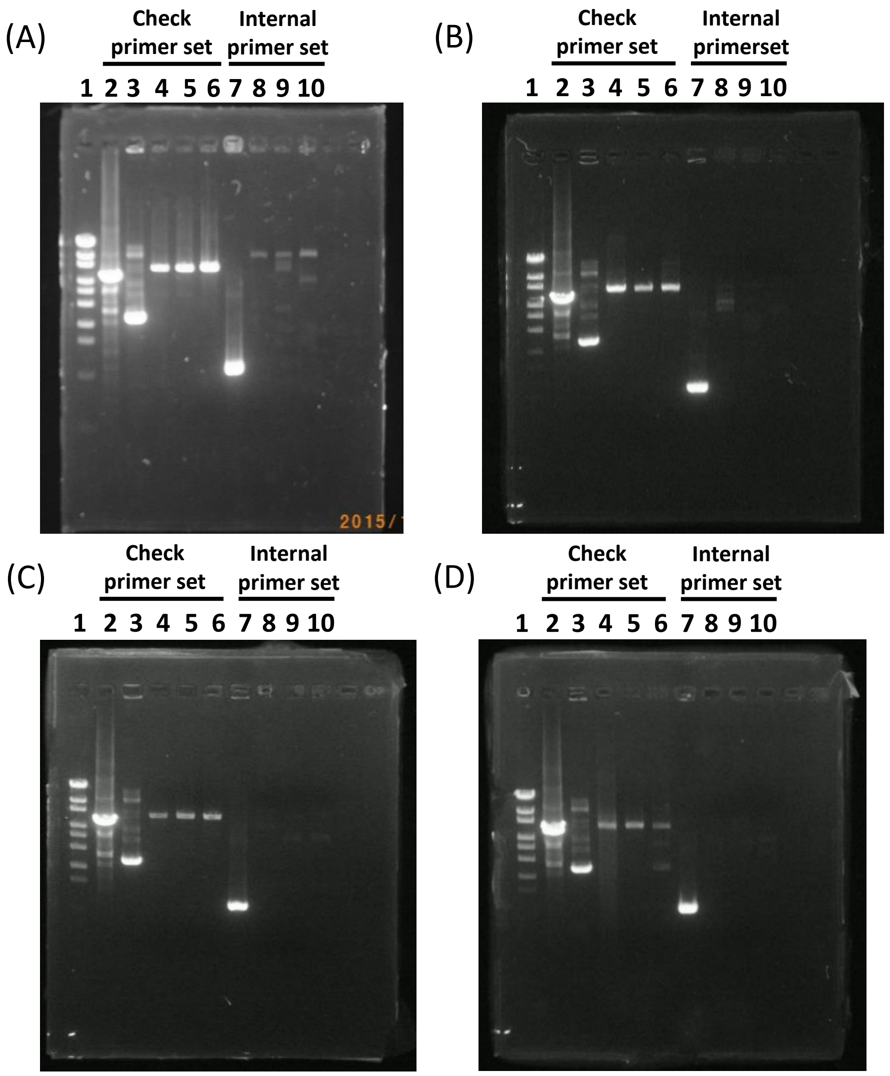

Fig. S12 Confirmation of strains with envD mutations using PCR

(A) envD-R1; (B) envD-R2; (C) envD-R3; (D) envD-R4

Lanes 2 to 6 and 7 to 10 are PCR products amplified using the check and internal primer sets, respectively. Lane 1 is DNA size maker of λStyI (see Fig. S8 for size distribution). Lane 2 is PCR products from the parental strain. Lanes 3 and 7 are PCR products from the Δ*envD* strain. Lanes 4 to 6 and 8 to 10 are PCR products from the constructed strains with envD mutations derived from the evolved strains.

Construction of the ΔenvD/ΔmcpA strain

The *ΔenvD/ΔmcpA* strain was constructed by replacing *mcpA* with a streptomycin resistance gene in the *ΔenvD* strain. The upstream and downstream regions of *envD* were also amplified with PCR using the envD_up_F and envD_up_R_Km, and

envD_down_F_Km and envD_down_R primer sets, respectively. The upstream region of *envD*, the kanamycin resistance gene, and the downstream regions of *envD* were connected with OE-PCR using the envD_up_F and envD_down_R primer set. The resulting PCR product was introduced into the parental strain. The transformants were screened on BG11 plates containing 20 μg/L of kanamycin. Complete segregation of *envD* by kanamycin resistance gene was confirmed by the disparity in the sizes of the PCR products generated using the envD_check_F and envD_check_R (check primer set), and envD_int_F and envD_check_R (internal primer set).

The result of agarose gel electrophoresis is shown in Fig. S13. In the PCR using the internal primer set, an approximately 1000 bp PCR product was observed in the parental strain (lane 2), which was absent in the Δ*envD* strains (lanes 3 to 5). In the PCR with the check primer set, approximately 5000 bp PCR product was observed in the parental strain (lane 6), whereas an approximately 2200 bp PCR product was observed in the Δ*mcpA* strains (lanes 7 to 9). These results are consistent with the fact that *envD* was replaced with a kanamycin resistance gene.

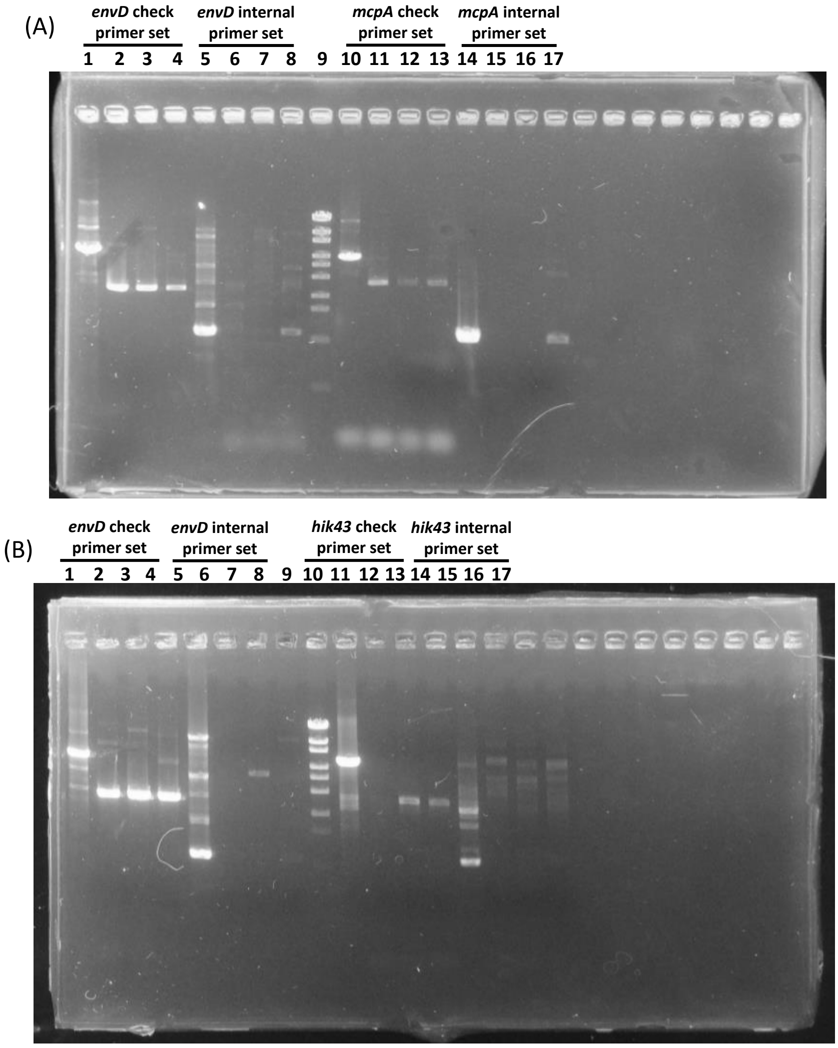

Fig. S13 Confirmation of double deletion strains using PCR

(A) Δ*envD*/Δ*mcpA*; (B) Δ*envD*/Δ*hik43*

Lanes 1 to 4 and 5 to 8 are PCR products amplified using the *envD* check and *envD* internal primer sets, respectively. Lane 9 is the DNA size maker of λStyI (see Fig. S8 for size distribution). Lines 10 to 17 are PCR products amplified using the check and internal primer sets for *mcpA* or *hik43*, respectively. Lanes 1, 5, 10, and 14 are PCR products from the parental strain. Lanes 2 to 4, 6 to 8, 11 to 13, and 15-17 are PCR products from the Δ*envD*/Δ*mcpA* or Δ*envD*/Δ*hik43* strains.

Construction of SE and SE-T(1) strains

SE and SE-T(1) strains, were constructed by introducing heterologous genes for *pdc* and *adhII* from *Zymomonas mobilis* into the *ndhB* site of parental and T(1) strains. A plasmid containing *pdc* and *adhII* placed between the upstream and downstream

sequences of the *ndhB* gene was constructed as previously reported (Yoshikawa et al., 2014). The plasmid was introduced into the parental and T(1) strains. The transformants were screened on BG11 plates containing 5 μg/L of ampicillin. Introduction of *pdc* and *adhII* genes was confirmed by the disparity in the sizes of the PCR products generated using ndhB_check_F and ndhB_check_R primer set.

The result of agarose gel electrophoresis is shown in Fig. S14. In the PCR using the check primer set, an approximately 1200 bp PCR product was observed in the parental strain (lane 2), and a 6000 bp PCR product was observed in the SE (lanes 3 to 5) and SE-T(1) (lanes 6 to 8) strains. These results are consistent with the fact that *pdc* and *adhII* genes were inserted into *ndhB* site of the SE and SE-T(1) strains.

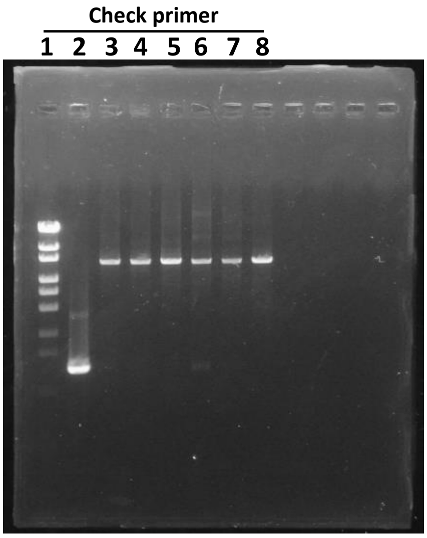

Fig. 14 Confirmation of introducing *pdc* and *adhII* genes into the parental and T(1) strains

Lane 1 is the DNA size maker of λStyI (see Fig. S8 for size distribution). Lanes 2 to 8 are PCR products amplified using the check primer set. Lanes 2, 3 to 5, and 6 to 8 are PCR products from the parental, SE, and SE-T(1) strains.

Table S1 List of primers used in this study

Primer	Sequence
mcpA_up_F	GAGTTTGCCCTGCCAGCGG
mcpA_up_R_Km	TCCCGTTGAATATGGCTCATTTTGCCGGTTTCCTCAGTCG
mcpA_down_F_Km	GATGCTCGATGAGTTTTTCTAAGGTCAGGGCAGTGTATTTCC
mcpA_down_R	CGGATCTGTCCTGAGCATATAC
hik43_up_F	CGCTAGCTATGAAACCCTGAC
hik43_up_R_Km	TCCCGTTGAATATGGCTCATTGCTTCTTGCTACTCTTCCAC
hik43_down_F_Km	GATGCTCGATGAGTTTTTCTAATGACAGAATCGTTGAGTCCC
hik43_down_R	CGGATCTAGGTCAGGATATTTCC
envD_up_F	TACTGCCGCCCAATTTACTG
envD_up_R_Km	TCCCGTTGAATATGGCTCATAAACCTGGGGGACGGCGGAAAG
envD_down_F_Km	GATGCTCGATGAGTTTTTCTAACAAGATTGGGGGCCAGCAACC
envD_down_R	AGGGGAAATTCCAGGGCATC
Km_F	ATGAGCCATATTCAACGGGA
Km_R	TTAGAAAAACTCATCGAGCATC
Sm_F	AGCGTAGCGACCGAGTGAG
Sm_R	TTATTTGCCGACTACCTTGGTG
mcpA_check_F	ACCATCAAACAACTCCTG
mcpA_check_R	TGGTTAAGCCCCAACTAC
mcpA_int_F	GCCATGGAAGAAGGTATC
hik43_check_F	CACCATGAGTTGGGAAGGGTGC
hik43_check_R	TCTAGATGGGCATGCCCTAG
hik43_int_F	AGGATGCCTGGGAAAAACTC
envD_check_F	CCTGTTAATCGGGAATTATTGG
envD_check_R	AAAGTTCCTGCTTGGATGCG
envD_int_F	TTTTGCGGCCAAGGAAAG
Km_int_F	TTGATGACGAGCGTAATGGC
mcpA_down_F_Sm	CCAAGGTAGTCGGCAAATAAGGTCAGGGCAGTGTATTTCC
mcpA_down_R_BglII	AGCAGATCTCGGATCTGTCCTGAGCATATAC
Sm_F_BglII	AGCAGATCTTTATTTGCCGACTACCTTGGTG
envD_down_F_Sm	CCAAGGTAGTCGGCAAATAACAAGATTGGGGGCCAGCAACC
envD_down_R_BglII	AGCAGATCTAGGGGAAATTCCAGGGCATC
ndhB_check_F	GTGAAGATGGTTACGCCAGC
ndhB_check_R	TCCTGGAGCCCTAAAGTTGC
